# Cerebrospinal fluid flow cytometry distinguishes psychosis spectrum disorders from differential diagnoses

**DOI:** 10.1038/s41380-021-01244-5

**Published:** 2021-08-06

**Authors:** Saskia Räuber, Michael Heming, Jonathan Repple, Tillmann Ruland, Rebecca Kuelby, Andreas Schulte-Mecklenbeck, Catharina C. Gross, Volker Arolt, Bernhard Baune, Tim Hahn, Udo Dannlowski, Sven G. Meuth, Nico Melzer, Heinz Wiendl, Gerd Meyer zu Hörste

**Affiliations:** 1 Department of Neurology with Institute of Translational Neurology, University of Münster, Münster, Germany; 2 Department of Neurology, Medical Faculty, Heinrich Heine University of Düsseldorf, Düsseldorf, Germany; 3 Department of Psychiatry, University of Münster, Münster, Germany; 4 Department of Psychiatry, Maria Brunn Hospital, Münster, Germany; 5 Department of Geriatrics, Helios Hospital Bonn, Bonn, Germany

**Keywords:** Diagnostic markers, Schizophrenia, Prognostic markers

## Abstract

Psychotic disorders are common and disabling mental conditions. The relative importance of immune-related mechanisms in psychotic disorders remains subject of debate. Here, we present a large-scale retrospective study of blood and cerebrospinal fluid (CSF) immune cell profiles of psychosis spectrum patients. We performed basic CSF analysis and multi-dimensional flow cytometry of CSF and blood cells from 59 patients with primary psychotic disorders (F20, F22, F23, and F25) in comparison to inflammatory (49 RRMS and 16 NMDARE patients) and non-inflammatory controls (52 IIH patients). We replicated the known expansion of monocytes in the blood of psychosis spectrum patients, that we identified to preferentially affect classical monocytes. In the CSF, we found a relative shift from lymphocytes to monocytes, increased protein levels, and evidence of blood–brain barrier disruption in psychosis. In fact, these CSF features confidently distinguished autoimmune encephalitis from psychosis despite similar (initial) clinical features. We then constructed machine learning models incorporating blood and CSF parameters and demonstrated their superior ability to differentiate psychosis from non-inflammatory controls compared to individual parameters. Multi-dimensional and multi-compartment immune cell signatures can thus support the diagnosis of psychosis spectrum disorders with the potential to accelerate diagnosis and initiation of therapy.

## Introduction

Psychosis spectrum disorders (henceforth termed psychotic disorders or psychosis for simplicity) are a heterogenous clinical entity associated with altered thoughts, perceptions, mood, and behavior [1]. Hallucinations, delusions, and thought disorganization are some of the typical clinical presentations [2]. The most common type of psychotic disorders is schizophrenia with a lifetime prevalence of up to 4.0 per 1000 persons [3]. Psychotic disorders often have a severe negative impact on patients’ personal, social, and occupational well-being [1]. In addition, schizophrenia imposes the highest financial burden on health care systems among all psychiatric diseases [4].

The etiology of psychotic disorders is thought to be multifactorial with both genetic and environmental factors contributing. However the exact pathophysiological mechanisms remain poorly understood [5–8]. One of the mechanisms inducing psychotic symptoms is likely an excess of dopamine or excessive activation of dopamine signaling. This widely accepted “dopamine hypothesis” is supported—among others—by the successful use of dopamine antagonists in the treatment of psychotic disorders [9].

Some evidence also indicates that the immune system contributes to the pathogenesis of psychotic spectrum disorders (henceforth termed psychosis for simplicity). Evidence supporting such an “immune hypothesis” was first reported decades ago [10]. More recently, polymorphisms near B lymphocyte enhancer genes, in major histocompatibility loci, encoding antigen presenting molecules [11], and in genes affecting the complement system (*C4A*, *C4B*) [12] were linked to an increased genetic risk of psychosis [13]. In addition, cell counts of neutrophils, monocytes, and natural killer (NK) cells have been reported to increase in the blood of patients with psychotic disorders and high neutrophil and monocyte counts were associated with disease severity [14–17]. However, data on the peripheral immune cell composition of psychosis spectrum patients, assessed by multidimensional flow cytometry (mFC), remain limited [15, 18]. Also, comparisons with inflammatory diseases of the brain that can clinically resemble psychosis have not been performed so far. Some imaging and post-mortem studies further support a function of immune cells in psychiatric diseases. Higher microglia counts and increased numbers of activated microglia were identified in patients with schizophrenia, pointing towards an involvement of the monocyte/macrophage system [19–21]. Although conceptually interesting, the utility of these parameters to support the differential diagnosis of psychosis remains unknown.

Cerebrospinal fluid (CSF) analysis serves as a routine diagnostic tool to identify inflammatory processes of the central nervous system (CNS). However, CSF analysis in psychosis usually only helps to exclude differential diagnoses but does not directly support the diagnosis. In fact, studies on the CSF immune cell profile of psychiatric patients are very scarce [22, 23]. Here, we performed a large-scale retrospective study on the blood and CSF immune cell profile of psychosis spectrum patients using mFC and machine learning algorithms. We investigated differences in immune cell profiles of primary psychotic patients and non-inflammatory as well as inflammatory controls. Applying different machine learning models, we also established the ability of mFC to support the diagnostic workup of psychotic disorders.

## Methods

### Patients

All CSF samples collected at the University Clinic Münster are processed in a centralized and specialized CSF lab and all CSF samples collected during regular working hours are processed with a standardized flow cytometry panel. We retrospectively screened the local clinical patient database at the University Clinic Münster, Germany to identify patients with available CSF mFC data *and* primary psychotic disorders encompassing schizophrenia (F20), delusional disorders (F22), acute and transient psychotic disorders (F23) or schizoaffective disorders (F25) based on the ICD-10 diagnostic criteria [24, 25]. All patients had been admitted to an inpatient unit of the Department of Psychiatry at the University Clinic Münster, Germany, between 2013 and 2018 and had received blood and CSF analysis including mFC as part of the clinical routine workup recommended to all patients presenting with psychotic symptoms in Germany [26]. Routine blood tests (blood cell count, renal-, liver-, thyroid function, and C-reactive protein) had been performed in all patients. In total, 62 patients were initially identified in the database that fulfilled the inclusion criteria and had mFC data available. Out of these, three patients were excluded based on the following exclusion criteria:

1. Signs of systemic infection.
2. Suspected viral encephalitis and bacterial meningitis assessed by an elevated CSF cell count (>5/µl), detection of viruses (Herpes simplex virus-1/2 [HSV-1/HSV-2], Cytomegalovirus [CMV], Epstein-Barr virus [EBV], Varicella zoster virus [VZV], Human herpesvirus-6 [HHV-6], and Enterovirus) in the CSF by polymerase-chain-reaction or bacteria by gram staining.

None of the initially identified patients had detectable anti-neuronal antibodies in serum and/or CSF assessed by immunofluorescence assays and immunoblotting (including Hu, Ri, ANNA-3, Yo, Ma/Ta, GAD65, Amphiphysin, NMDA receptor, GABA-a/b receptor, LGI1, CASPR2, glycine receptor, mGluR1/5, and AMPA receptor antibodies). In total, 59 patients were included in the final analysis and are referred to as the F2x group throughout the manuscript (Supplementary Table 1).

We retrospectively identified three control cohorts for comparison: (1) idiopathic intracranial hypertension (IIH; ICD-10 G93.2; *n* = 52), a disease characterized by excessive CSF causing headache and visual disturbances, but without inflammation or CSF abnormalities [27], (2) treatment-naive patients with a first diagnosis of relapsing-remitting multiple sclerosis (RRMS; ICD-10 G35.1; *n* = 49), an autoimmune disease of the CNS characterized by bouts of inflammatory demyelination and neurological disability [28]; (3) autoimmune encephalitis associated with CSF antibodies targeting the NMDA receptor (NMDARE; ICD-10 G13.1; *n* = 16), a disease that can cause symptoms similar to primary psychosis [29]. All patients were admitted to the Department of Neurology with Institute of Translational Neurology at the University Clinic Münster, Germany, between 2012 and 2019. None of the control patients had been diagnosed with a psychotic disorder. Psychiatric comorbidities were noted in ten control patients (eight depression, one borderline personality disorder, one anxiety disorder). The IIH and RRMS cohorts had been “used” and described previously [30].

RRMS patients were first diagnosed at time of sample taking—based on the revised McDonald criteria [31, 32]—and were treatment naive. In order to exclude differential diagnoses, a broad diagnostic workup was performed as described previously [33]. Patients with IIH met the diagnostic criteria of IIH including an elevated opening pressure (>200 mmH_2_O) on lumbar puncture, symptoms indicative of IIH, normal routine CSF parameters and absence of structural abnormalities in cranial MR imaging [27]. Patients with NMDARE were diagnosed according to the diagnostic criteria defined previously: a rapid onset (<3 month) of four out of six typical clinical symptoms, either abnormal EEG or pleocytosis/oligoclonal bands in CSF studies, exclusion of differential diagnoses and detection of CSF-IgG antibodies targeting the GluN1 subunit of the NMDA receptor [29].

The study was conducted according to the Declaration of Helsinki. All data were collected during clinical routine workup. Anonymization was performed and data were reanalyzed retrospectively. Sample collection and analysis of biomaterial was approved by the Ethics Committee of the Board of Physicians of the Region Westfalen-Lippe and of the Westfälische Wilhelms-University Münster (reference number: 2019-712-f-S). Samples were solely collected as part of the clinical routine workup and anonymization was performed, thus, in accordance with the German law, no written consent was required.

### Quantification of the severity and duration of psychosis

Disease severity and impact of the psychiatric disease on social and occupational life was assessed by the Global Assessment of Functioning (GAF) scale. The GAF was rated by an experienced psychiatrist on admission. The scale consists of 100 points divided into 10-point intervals with 0–10 being the lowest score (severely ill) and 90–100 presenting the highest score (the healthiest) [34]. In order to analyze and compare the immune cell profiles at different disease stages, F2x patients were categorized into subgroups based on the duration of disease: 1. first diagnosis, 2. short disease course (1–5 years), 3. long disease course (>5 years). Duration of disease was defined as time between initial manifestation and sample taking.

### Basic CSF characteristics

Lumbar puncture was performed under sterile conditions and samples were processed within 1 hour to assure optimal sample quality. CSF cells were counted in a Fuchs-Rosenthal chamber. Nephelometry was used to assess protein concentrations and immunoglobulin levels (IgG, IgA, and IgM). Serum/CSF protein and immunoglobulin levels were compared and a Reiber scheme was created to evaluate the integrity of the blood-CSF barrier (BCSFB) [35]. For simplicity, BCSFB dysfunction is termed blood–brain barrier disruption (BBBD) throughout the manuscript. Isoelectric focusing and silver nitrate staining was performed to detect oligoclonal bands (ocbs) [36].

### Flow cytometry

CSF and blood samples were analyzed in the centralized CSF laboratory of the University Clinic Münster within 1 h of sample taking. Standardized mFC of CSF and blood samples using a predefined staining panel are routinely performed at our center of all CSF samples collected during regular working hours. Analysis was performed once per sample.

Samples were centrifuged for 15 min at 300 × *g*, supernatant was removed, and staining was performed based on an established protocol [37]. The following antibodies were used: CD3 (UCHT1); CD4 (13B8.2); CD8 (B9.11); CD14 (RMO52); CD16 (3G8); CD19 (J3–119); CD45 (J.33); CD56 (C218); CD138 (B-A38); HLA-DR (Immu-357) (all Beckman Coulter, clones in brackets). Gating was performed on forward scatter and sideward scatter and afterward on CD45+ cells. Percentage of cell population was analyzed as described before [30]. Analysis was performed using a Navious flow cytometer (Beckmann Coulter) and the software *Kaluza* (version 2.1).

### Data analysis

The software *R* (version 4.0) was used for data analysis [38]. If the dependent variable was continuous, statistical significance was determined using the Kruskal Wallis test with the two-sided post hoc Dunn test (multiple groups) or two-sided Mann–Whitney *U* test (two groups). If the dependent variable was categorial, we used the two-sided Fisher test (pairwise comparisons with the *R* package *RVAideMemoire* for multiple groups). *P* values were adjusted for multiple hypothesis testing using Benjamini-Hochberg’s procedure in the case of multiple group comparisons. A *p* value of <0.05 was regarded as statistically significant. To adjust for sex and age, we performed a multiple linear regression analysis using *R*. We performed principal component analysis (PCA) with the *R* package FactorMineR as described previously [30, 39]. Data were scaled in advance. Heatmaps were created with the R package *pheatmap*. In order to create heatmaps, data were grouped by disease and the mean of each parameter was calculated. Next, data were scaled row wise. Finally, the results were visualized in a heatmap using the *R* package *pheatmap*. Rows were clustered hierarchically using euclidean distance measure and complete linkage.

The R package *pROC* was used to perform receiver operating characteristic (ROC) analysis. ROC analysis evaluates the performance of a classification based on sensitivity and specificity of a test represented by the area under the curve (AUC). AUC values range from 0.5 (uninformative) to an AUC of 1 (perfect). The correlation scatter plots were created with the *ggscatter R* package with Pearson correlation coefficient.

### Machine learning

The following machine learning models were tested with the *R* package *caret*: Linear discriminant analysis, ridge regression, lasso regression, elastic net regression, support vector machines, Naive Bayes, Flexible discriminant analysis, and logistic regression. Data were preprocessed by centering, scaling and BoxCox transformation. Furthermore, near zero variance predictors and highly correlated predictors (>0.9) were removed. We performed recursive feature elimination (RFE) on all algorithms that do not have an inherent feature selection with the number of parameters ranging from one to 20. Because of the small sample size, we performed tenfold cross validation with five repeats. In the case of RFE, we carried out nested cross validation: an outer loop to conduct feature selection and an inner loop to optimize the tuning parameters. We used the distance from perfect sensitivity (1) and perfect specificity (1) as the performance metric, aiming to minimize this distance. Variable importance was calculated with the varImpPlot function in *caret*. F2x was defined as positive and IIH as negative.

## Results

### Retrospective identification of psychosis and inflammatory and non-inflammatory control patients

**Fig. 1:**
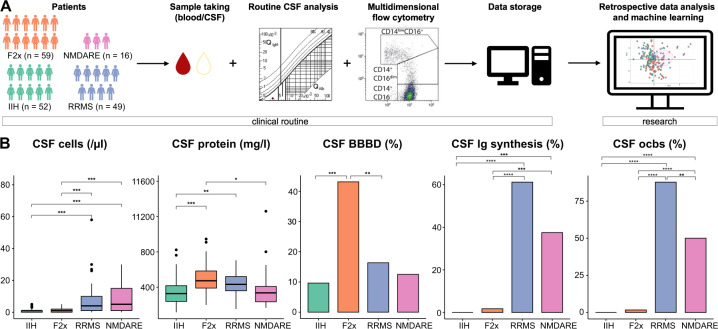
Basic CSF characteristics are in accordance with expectations for F2x patients and controls. **A** Illustration of study design **B** Basic CSF characteristics: cells were counted manually in a Fuchs-Rosenthal chamber; protein was assessed by nephelometry; BBBD was evaluated based on the serum/CSF albumin ratio; Ig synthesis was assessed by nephelometry; oligoclonal bands were detected by isoelectric focusing and silver nitrate staining. Box plots show the lower quartile, median and upper quartile. Whiskers depict 1.5 times the interquartile range of the box and outliers are illustrated by dots. Significance was calculated with the Kruskal Wallis test and post hoc two-sided Dunn test if the dependent variable was continuous. Fisher test was carried out for categorical dependent variables. *P* values were adjusted for multiple hypothesis testing with Benjamini-Hochberg’s procedure. (BBBD: blood-brain-barrier dysfunction; CSF: cerebrospinal fluid; F2x: patients with psychotic disorder, Ig: immunoglobulin; IIH: intracranial hypertension; NMDARE: anti-NMDA-receptor encephalitis; ocb: oligoclonal band; RRMS: Relapsing-Remitting Multiple Sclerosis).

The immune system may contribute to psychosis and the CSF provides a unique “diagnostic window” into immune mechanisms of the brain. We therefore retrospectively queried the local clinical database for patients who had been admitted with a diagnosis of primary psychosis (based on the ICD-10 diagnostic criteria [24, 25]) and had received CSF analysis, including mFC (Fig. 1A, methods).

In total, 59 patients (schizophrenia [F20, *n* = 34/57.6%], delusional disorders [F22, *n* = 6/10.2%], acute and transient psychotic disorders [F23.0, *n* = 8/13.6%], and schizoaffective disorders [F25, *n* = 11/18.6%]) were included and are henceforth collectively referred to as the F2x or psychosis group (Table 1). Approximately one third of the F2x patients (33.9%) were first diagnosed with psychosis, 81.4% had positive psychotic symptoms and 93.2% of patients were treated with antipsychotic drugs at the time of sampling (Table 1, Supplementary Table 1). Average time from first diagnosis until sampling was 4.9 ± 9.4 years and from admission until sampling was 28.6 ± 31.1 days (Supplementary Table 1). None of the patients had received any immunosuppressants or immunomodulatory treatment. Patients with IIH (*n* = 52), RRMS (*n* = 49), and NMDARE (*n* = 16) served as non-inflammatory and inflammatory controls (Methods). NMDARE can cause symptoms similar to primary psychosis [29].

**Table 1:** Demographics and basic disease characteristics of F2x patients and controls.

	F2x	IIH	RRMS	NMDARE
Number of patients (F20/22/23/25) [number/%]	59 (34/57.6; 6/10.2; 8/13.6; 11/18.6)	52	49	16
Age (median with range) [years]	31.0 (18–66)	33.7 (18–66)	34.9 (18–55)	37.0 (18–78)
Male:female	1.68:1	0.24:1	0.48:1	0.33:1
Pt. first diagnosed [number/%]	20/33.9	na	49/100	15/93.8
Positive psychotic symptoms (at sample taking) [number/%]	48/81.4	0/0	0/0	10/62.5
Antipsychotic drugs [number/%]	55/93.2	na	na	na
Anti-NMDA receptor ab [number/%]	0/0	na	na	16/100

*Ab* antibody, *F2x* patients with psychotic disorder, *IIH* intracranial hypertension, *NMDARE* anti-NMDA-receptor encephalitis, *Pt* patients, *RRMS* Relapsing-Remitting Multiple Sclerosis.

The age was well matched between groups and the average age of F2x patients was 31.0 years (Table 1). Sex was unbalanced towards male in the F2x group (m:f = 1.68:1) and towards female in the control groups (m:f = IIH: 0.24:1; RRMS: 0.48:1; NMDARE 0.33:1) (Table 1). Clinical data of F2x and of NMDARE patients are summarized in Supplementary Table 1. We thus successfully composed large and relatively well-matched cohorts of psychosis spectrum patients and inflammatory and non-inflammatory controls.

### Basic CSF parameters partially distinguish psychosis from controls

Previous studies have reported CSF abnormalities in psychosis spectrum patients (e.g., elevated total protein concentration, and albumin ratio) [40–42] and we first aimed to replicate these findings. We therefore analyzed whether basic CSF parameters differed between F2x and IIH patients and added RRMS and NMDARE patients as unique inflammatory controls.

We assessed basic CSF parameters (total cell count, protein concentration, serum/CSF albumin ratio, intrathecal immunoglobulin synthesis [Ig synthesis], oligoclonal bands [ocbs]) and found that total CSF cell counts, Ig synthesis, and ocbs were significantly higher in the RRMS and NMDARE groups compared to the IIH and F2x groups (Fig. 1B). These changes are consistent with known acute inflammatory changes in both diseases [43, 44] and thus support the validity of our approach. When comparing F2x patients with controls, we found that the CSF protein concentration was elevated in the CSF of F2x patients (492.5 mg/l) in relation to IIH (356.2 mg/l) and NMDARE (410.8 mg/l) patients, but not to RRMS patients (436.0 mg/l) (Fig. 1B). Also, blood-brain barrier disruption (BBBD) defined by an elevated CSF/blood albumin ratio was significantly more frequent in F2x patients (43.5%) than in controls (Fig. 1B). To account for the heterogeneity of the F2x group (Table 1 and Supplementary Table 1), we next categorized F2x patients based on disease duration into 1) first diagnosis, 2) short disease (1–5 years), and 3) long disease (>5 years). Basic CSF parameters were not significantly different between groups (Supplementary Fig. 1A). Basic CSF parameters, except for Ig synthesis and ocbs, also did not significantly differ between patients with and without positive psychotic symptoms at the time of CSF analysis (Supplementary Fig. 1B). In accordance with previous findings [41], psychosis thus exhibited signs of BBB disruption associated with excess CSF protein.

### Psychosis features a specific intermediate-to-classic phenotypic shift of monocytes in the blood

**Fig. 2:**
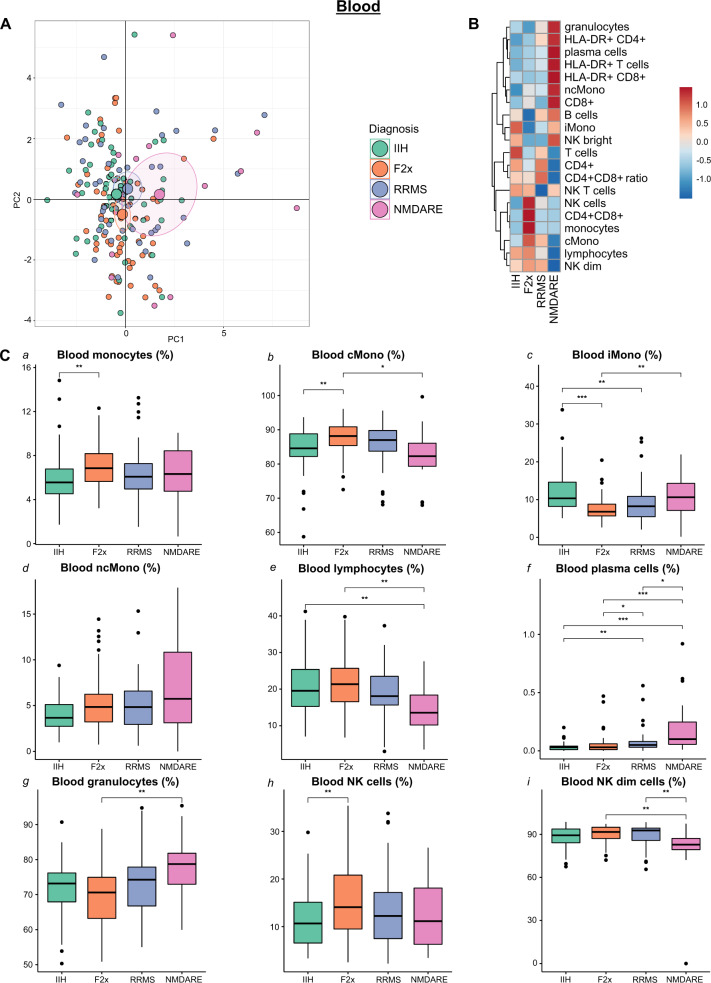
Blood monocyte subpopulations distinguish F2x from control patients. **A** PCA of blood flow cytometry parameters: Each patient is depicted as a multidimensional data point. The group means are illustrated as larger circles and the confidence intervals are shown by ellipses around each group mean point. **B** Heatmap of blood flow cytometry parameters: The mean of each parameter was calculated, scaled, centered, and clustered hierarchically. **C** Box plots of selected individual blood flow cytometry parameters: Lower quartile, median and upper quartile are shown by boxes. Whiskers depict 1.5 times the interquartile range of the box and outliers are illustrated by dots. The Kruskal Wallis tests with post hoc two-sided Dunn test and Benjamini-Hochberg’s adjusted *p* values were used to determine the significance. (cMono: classical monocytes; F2x: patients with psychotic disorder; IIH: intracranial hypertension; iMono: intermediate monocytes; NK: natural killer cells; ncMono: non-classical monocytes; NMDARE: anti-NMDA-receptor encephalitis; PC: principal component; PCA: principal component analysis; RRMS: Relapsing-Remitting Multiple Sclerosis).

Available mFC studies from psychosis spectrum patients remain limited to the peripheral blood (PB) [15] and lack comparison with inflammatory CNS diseases. We therefore collected blood mFC data from all patients in our study and first performed principal component analysis (PCA) to reduce the dimensionality and detect gross patterns (Fig. 2A). In this PB-based analysis, IIH and RRMS patients clustered together and were largely indistinguishable (Fig. 2A) in accordance with the absence of blood-based markers of MS. Principal component (PC) 2 tended to distinguish F2x from IIH and RRMS and this was driven by the parameters CD4+ cells, CD4+ CD8+ ratio, and intermediate monocytes (Supplementary Fig. 2A). In contrast, NMDARE patients were distinct from the other three groups (Fig. 2A), and this was mainly driven in PC1 by plasma cells, NK cells, HLA-DR+ T cells, and HLA-DR+ CD4+ cells (Supplementary Fig. 2A).

Plotting parameters individually confirmed significant alterations in the proportion of monocytes and in the composition of monocyte subsets in PB of F2x patients as well as an elevated proportion of NK cells (Fig. 2B, C). Compared to the IIH group, F2x patients featured higher proportions of monocytes and NK cells (Fig. 2C *a,* h). Monocytes are classified into subsets and classical monocytes (CD14+ CD16-; cMono) make up 80% of all monocytes, while non-classical (CD14low CD16hi; ncMono) and intermediate monocytes (CD14+ CD16+ ; iMono) are less frequent in PB [45]. Subset analysis revealed a reduction of iMono and inversely an increase of cMono in F2x in relation to IIH and NMDARE patients (Fig. 2C *b*, *c*).

As (initial) clinical presentation of NMDARE can resemble primary psychotic disorders [29] we focused on comparing the PB of NMDARE with F2x patients because available data are limited [46]. This revealed a higher proportion of plasma cells and granulocytes and a decrease in NK dim cells in the PB of NMDARE compared to F2x patients (Fig. 2C *f*, *g*, *i*). All significant blood parameters, except differences in total monocytes between F2x and IIH patients and plasma cells between F2x and MS patients, remained significant after adjusting for age and sex using multiple regression analysis (Supplementary Table 2). Subgroup analyses of F2x patients (Methods) also returned no significant differences between F2x patient groups of variable disease duration (Supplementary Fig. 3). When comparing F2x patients with and without positive psychotic symptoms, NK cells and CD8+ were significantly higher and CD4+, CD4+ CD8+ ratio as well as T cells significantly lower in F2x patients with positive psychotic symptoms. No differences were observed in cells of the monocyte lineage (Supplementary Fig. 4). Overall, we found that the known expansion of blood monocytes in psychosis preferentially affected cMono and was maintained when controlling for confounders and disease heterogeneity.

### Non-classical monocytes expand in the CSF of psychosis spectrum patients

**Fig. 3:**
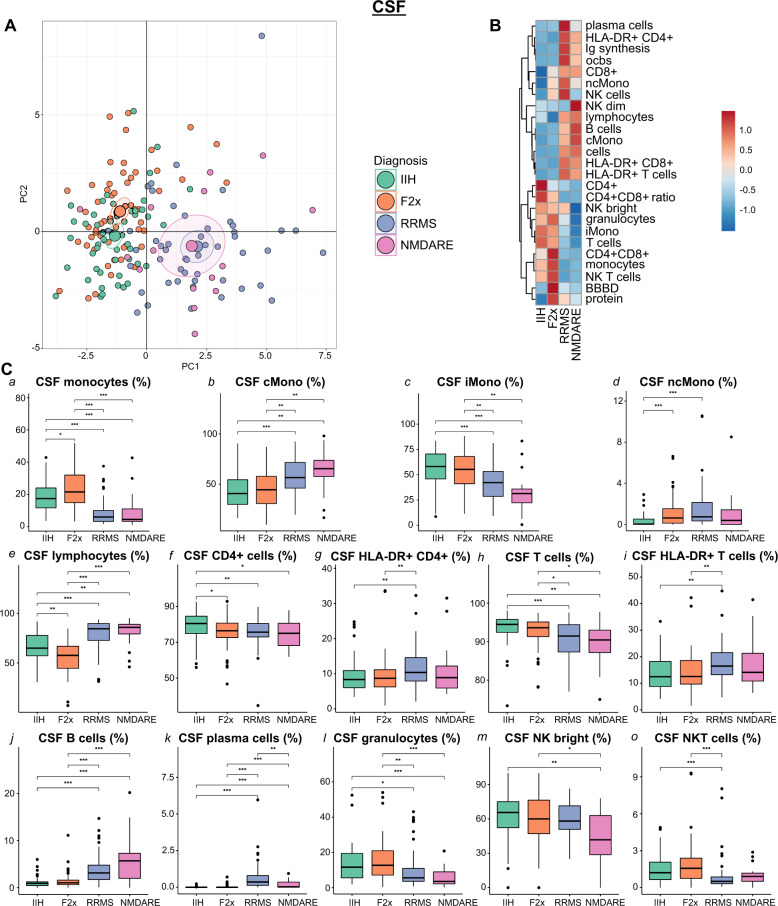
A unique pattern of CSF leukocytes differentiates F2x patients from diverse control patients. **A** PCA of CSF basic and flow cytometry parameters: Each patient is depicted as a multidimensional data point. The group means are illustrated as larger circles and the confidence intervals are shown by ellipses around each group mean point. **B** Heatmap of CSF basic and flow cytometry parameters: The mean of each parameter was calculated, scaled, centered, and clustered hierarchically. **C** Box plots of selected individual CSF flow cytometry parameters: Lower quartile, median and upper quartile are shown by boxes. Whiskers depict 1.5 times the interquartile range of the box and outliers are illustrated by dots. The Kruskal Wallis tests with post hoc two-sided Dunn test and Benjamini-Hochberg’s adjusted *p* values were used to calculate the significance. (BBBD: blood-brain-barrier dysfunction; cMono: classical monocytes; CSF: cerebrospinal fluid; F2x: patients with psychotic disorder; Ig: immunoglobulin; IIH: intracranial hypertension; iMono: intermediate monocytes; NK: natural killer cells; ncMono: non-classical monocytes; NMDARE: anti-NMDA-receptor encephalitis; ocb: oligoclonal band; PC: principal component; PCA: principal component analysis; RRMS: Relapsing-Remitting Multiple Sclerosis).

We next translated our mFC approach to the CSF. We integrated basic CSF parameters with mFC-derived CSF parameters from all patients in our study and again performed PCA. In this analysis, NMDARE patients clearly separated from F2x and IIH patients and showed an overlap with RRMS patients (Fig. 3A). The main contributors to this PC1 were B cells, Ig synthesis, plasma cells, ocbs, and total cell count indicating shared signs of B cell-driven inflammation in the CSF in RRMS and NMDARE (Supplementary Fig. 2B). Distinction between F2x and IIH samples along PC2 was slightly more pronounced than in the blood (Fig. 3A) and was driven preferentially by total monocytes and CD8+ T cells (Supplementary Fig. 2B). This indicated a unique pattern of cellular changes in the CSF in psychosis.

Plotting individual parameters (Fig. 3B, C) emphasized that the proportion of total monocytes was higher in the F2x group, while lymphocytes were less frequent compared to controls (Fig. 3C a*, e*). Unlike in the blood, this lymphocyte-to-monocyte shift was mainly driven by iMono and ncMono (Fig. 3C *c, d*). Notably, the proportions of monocytes, iMono, and granulocytes were higher in F2x in comparison to NMDARE and RRMS patients (Fig. 3C *a, c, l*). NcMono increased in the F2x compared to the IIH group (Fig. 3C *d*). When comparing F2x to NMDARE, we observed an increase in lymphocytes, especially B cells and plasma cells, in the CSF of NMDARE in relation to F2x patients (Fig. 3C *e, j, k*). We further identified differences in monocytes and monocyte subpopulations, including cMono and iMono, in the CSF of NMDARE patients in comparison to F2x patients and to non-inflammatory controls (Fig. 3C *a*–*c*). All CSF changes, except differences of CD4+ T cells between F2x and IIH patients and of plasma cells, BBBD, and protein between F2x and NMDARE patients, remained statistically significant when correcting for sex and age (Supplementary Table 2). Moreover, subsetting F2x patients based on disease duration did not reveal significant differences in CSF immune cell profiles between disease stages (Supplementary Fig. 5). Comparison of CSF immune cell profiles of F2x patients with and without positive psychotic symptoms identified higher proportions of monocytes, NKT cells, and NK bright cells in F2x patients with positive psychotic symptoms while monocytes subpopulations did not differ (Supplementary Fig. 6A). In summary, this indicated that the lymphocyte-to-monocyte shift in psychosis was maintained across immunological compartments; albeit with a location-specific non-classical phenotype in the CSF.

### Non-classical monocytes correlate with psychosis severity

**Fig. 4:**
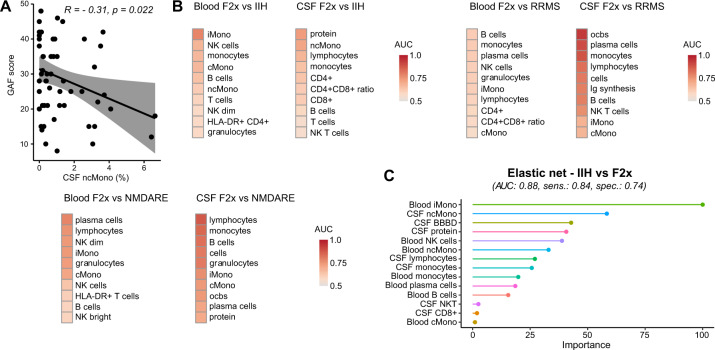
Non-classical monocytes in CSF correlate with psychosis severity and multiparametric models distinguish F2x from control patients. **A** Disease severity was assessed by the GAF. Correlation analysis was performed with the Pearson correlation coefficient (R) and linear regression analysis. The gray areas show the confidence interval. **B** ROC analysis of F2x, IIH, RRMS and NMDARE patients with basic CSF, blood, and CSF flow cytometry parameters: AUC values were calculated, sorted by value, and depicted in a heatmap. **C** Blood and CSF parameters were combined, and the most powerful parameters were identified by different machine learning approaches. Feature selection methods were applied to reduce the numbers of predictors. We used the distance metric from perfect sensitivity and specificity as the performance metric. Different models were trained to minimize the distance and the final models were benchmarked based on AUC, sensitivity, and specificity (Supplementary Fig. 4). The top performing machine learning approaches were chosen. The variable importance of the best performing machine learning approaches are shown. When comparing IIH to F2x, F2x was defined as positive and IIH as negative. (AUC: area under the curve; BBBD: blood-brain-barrier dysfunction; cMono: classical monocytes; CSF: cerebrospinal fluid; F2x: patients with psychotic disorder; Ig: immunoglobulin; IIH: intracranial hypertension; iMono: intermediate monocytes; LDA RFE: recursive feature elimination based on linear discriminant analysis; NB: naive bayes; NK: natural killer cells; ncMono: non-classical monocytes; NMDARE: anti-NMDA-receptor encephalitis; ocb: oligoclonal band; ROC: receiver operating characteristic; RRMS: Relapsing-Remitting Multiple Sclerosis; Sens: sensitivity; Spec: specificity).

We next investigated potential immunological determinants of the severity of psychosis. We therefore tested for correlations between psychosis severity, quantified by the Global Assessment of Functioning (GAF) scale, and all collected blood and CSF parameters. We found that the proportions of CSF ncMono, PB T cells and PB CD8+ lymphocytes negatively correlated and NK cells in PB positively correlated with the GAF (Fig. 4A and Supplementary Fig. 6B). Low GAF indicates a high psychosis severity. Furthermore, we assessed previous hospitalizations due to psychotic symptoms for every F2x patient and also performed correlation analysis. There was a negative correlation between the total number of previous hospitalizations due to psychotic symptoms and NK cells in PB and a positive correlation with T cells and NK bright in PB (Supplementary Fig. 6B). Quantifying monocytes in the CSF and NK cells in the blood might thus have potential as a severity marker.

### Multidimensional flow cytometry can support the diagnostic workup of psychosis

NMDARE may initially present with psychosis [47] emphasizing the need for diagnostic tools. We therefore performed ROC analysis and calculated the AUC to quantify the capacity of single parameters in distinguishing psychosis from other diseases. Best discriminators of the F2x versus IIH comparison were iMono in PB and protein and ncMono in CSF (Fig. 4B, Supplementary Table 3). As expected for RRMS, ocbs, followed by plasma cells, and monocytes in the CSF differentiated F2x from RRMS (Fig. 4B, Supplementary Table 3). When comparing F2x to NMDARE, lymphocytes, monocytes, and B cells in the CSF and plasma cells in the PB were the parameters with highest discriminatory power (Fig. 4B, Supplementary Table 3).

We next combined all blood and CSF parameters and applied machine learning approaches to identify the most powerful discriminatory parameters based on the AUC value. We applied feature selection methods, such as RFE or inherent feature selection mechanism of the algorithm, to reduce the numbers of predictors to make the model less complex and more interpretable. This represents an unbiased approach to select the most relevant features of the models (Methods).

The elastic net approach achieved the highest AUC value (0.88) when distinguishing between F2x and IIH (Supplementary Fig. 6C, Supplementary Table 4). This elastic net included 14 predictors, of which PB iMono and CSF ncMono had the highest variable importance (Fig. 4C). PB and CSF mFC parameters can thus support the rapid differential diagnosis of psychosis and machine learning methods showed superior discriminatory ability compared to individual parameters.

## Discussion

In this study, we collected a large dataset of highly mFC data from the blood and CSF aiming to better understand immune mechanisms in psychosis. We identified a characteristic immune cell profile of primary psychotic disorders showing compartment-specific alterations specifically in myeloid cell subsets and distinct from non-inflammatory controls and classical inflammatory CNS diseases. We replicated impaired barrier function and an increase in blood monocytes in psychosis and observed that the monocyte expansion in psychosis preferentially affected cMono, while ncMono increased in the CSF. NcMono in the CSF also correlated with disease severity. We quantitatively benchmarked single parameters and models with multiple parameters for their discriminatory ability and found superiority of multi-parameters models compared to single parameters. We thus provided evidence for the diagnostic and prognostic potential of immunological analyses in psychosis in support of the “immune hypothesis” in psychosis.

Our study features several unique inflammatory and non-inflammatory control groups [27–29] and exceeds previous studies in several ways. It emphasizes the potential of blood analysis to support diagnostic work-up in psychosis. Leukocytes in the PB of psychosis spectrum patients had been analyzed previously [14–18], but CSF data remained scarce [22, 23]. The close vicinity of CSF to CNS tissue supports its suitability as an immediate surrogate of local pathology. In addition, inflammatory CNS diseases—especially with clinical similarity—were not previously tested in comparison to psychosis [14, 23] and did not apply state-of-the-art machine learning approaches.

Our study is limited by its retrospective design, small sample sizes, and some confounding factors. Whether our findings also allow predicting the prognosis of psychosis spectrum patients, will require prospective investigations. By using repeated cross-validation and low variance machine learning algorithms (e.g., regularized regression instead of deep neural networks), we attempted to avoid overfitting despite low sample size. Nonetheless, a larger independent test cohort would be necessary to validate the models. Patient numbers were too low to benchmark models to discriminate between F2x and NMDARE. We also used statistical approaches to account for patient heterogeneity and potential confounders which substantiated our main findings. We were unable to include purely healthy controls because of ethical concerns in performing an invasive lumbar puncture for only scientific reasons. Beyond that, F2x patients with elevated CSF cell counts were excluded to rule out secondary (infectious) causes of psychosis which could potentially bias our results. Due to the retrospective design, only the GAF was available as a surrogate of clinical functioning and psychosis severity. The lack of other clinical scores (f.ex SCID [48] and PANSS [49]) limits the generalizability of our study. In addition, the majority of patients were treated with antipsychotic drugs with confounding potential. Thus, future studies will need to assess immune cell profiles of treatment naive patients and include more detailed clinical characterization in a prospective design. Our data hint towards a disease-stage independent, trait-like pattern of abnormalities in the blood and CSF immune cell composition. Future prospective, longitudinal studies especially with well-characterized subjects at ultra-high risk for psychosis [50] will be required to address the unanswered question of state-vs-trait alterations of immune cell compositions in psychosis and its relation to differential disease trajectories and treatment effects.

Primary psychotic disorders present a heterogeneous clinical entity with complex pathophysiology [5–10]. Previous data indicate immune dysregulation and provide evidence for neuroinflammation in at least a subgroup of patients with primary psychotic disorders and these studies had applied blood analysis, MRI, histological studies, and routine CSF analysis [19–21, 51–54]. In line with previous observations [54, 55], we detected BBBD in primary psychosis spectrum patients. BBBD has been previously linked to neuroinflammation and oxidative stress in schizophrenia patients and animal models [54]. For example, loss or dysfunction of astroglia as regulators of cerebral blood flow and volume and BBB permeability have been noted in schizophrenia patients or patients at risk for psychosis [54, 56, 57]. In addition, microglial activation can cause neuronal injury and impair BBB function by induction of oxidative stress and release of pro-inflammatory cytokines [54]. TSPO-PET imaging and PET/MRI of patients with psychotic disorders detected higher numbers of activated myeloid cells in the grey matter [19, 20] and post-mortem brain tissue also revealed an increase in microglia density in schizophrenia patients compared to controls [21]. Consistent with a suggested dysregulation of *innate* immunity in the CNS, our PB analysis hints towards a contribution of myeloid cells to the pathogenesis of primary psychotic disorders [14, 19–21, 23, 58]. Elevated numbers of blood monocytes [14] and their higher inflammatory capacity was previously detected in schizophrenia patients [59]. Cytokine analyses also support the relevance of innate immunity in psychosis [51, 52, 60]. Cytokines released by activated myeloid cells (f. ex. IL-1, IL-6, IL-12) were elevated in a subset of psychosis spectrum patients [51, 61, 62]. In accordance, we identified the expansion of monocytes to affect classical vs. non-classical subsets in a compartment-specific fashion. Notably, monocytes acquire a partially microglia-like phenotype in the CSF [33, 63]. Although the relationship between CSF myeloid cells and tissue-resident microglia remains elusive, it is intriguing to speculate that CSF-resident myeloid cells mirror an inflammatory parenchymal pathology in psychosis. Overall, combining our data with results from previous studies, one may speculate that peripheral and intrathecal alterations in immune cell composition, especially in *innate* immunity, together with an impaired barrier function contribute to the local pathology in the CNS of patients with primary psychotic disorders in support of the immune hypothesis in psychosis.

Differentiating primary psychotic disorders from other medical conditions presenting with psychosis can be difficult [64]. NMDARE is an important differential diagnosis of primary psychosis [47]. NMDAR-antibodies cause reversible neuronal dysfunction, thus, early diagnosis and immunotherapy determine outcome [65, 66]. Detection of NMDAR-antibodies in CSF supports the diagnosis, however, lumbar puncture is an invasive procedure and is not part of the routine workup of primary psychotic disorders according to the current clinical guidelines in many countries [67]. Blood analysis is less invasive and can be performed quickly. We found that mFC of PB can differentiate primary from secondary psychotic disorders with high discriminatory ability only slightly inferior to CSF analysis. This could enable a rapid and less invasive prioritization of psychosis spectrum patients for further diagnostic workup in order to avoid diagnostic and therapeutic delay.

In summary, we here provide the first broad study on blood and CSF immune cell composition of patients with psychotic disorders in relation to non-inflammatory (IIH) and inflammatory (RRMS, NMDARE) controls. Alterations in peripheral and intrathecal myeloid compartments hint towards a contribution of the *innate* immune system to the multifactorial etiology of primary psychotic disorders. Thus, mFC might be a valuable tool in the diagnostic workup of psychotic disorders and could promote early diagnosis and treatment to improve outcomes in the future.

### Data sharing

Further information and requests for resources, anonymized clinical and flow cytometry data should be directed to and will be fulfilled by the Lead Contact, Gerd Meyer zu Hörste (gerd.meyerzuhoerste@ukmuenster.de).

## Supplementary information

Supplementary figures

Supplementary tables
