# Supplementary material for: Cerebrospinal fluid flow cytometry distinguishes psychosis spectrum disorders from differential diagnoses
Source: Mol Psychiatry. 2021 Aug 6;26(12):7661–70. doi: 10.1038/s41380-021-01244-5 (PMC8873003; doi:10.1038/s41380-021-01244-5)
Supplement: Supplementary file 1 — Supplementary figures [file 41380_2021_1244_MOESM1_ESM.pdf]

Supplementary figure 1

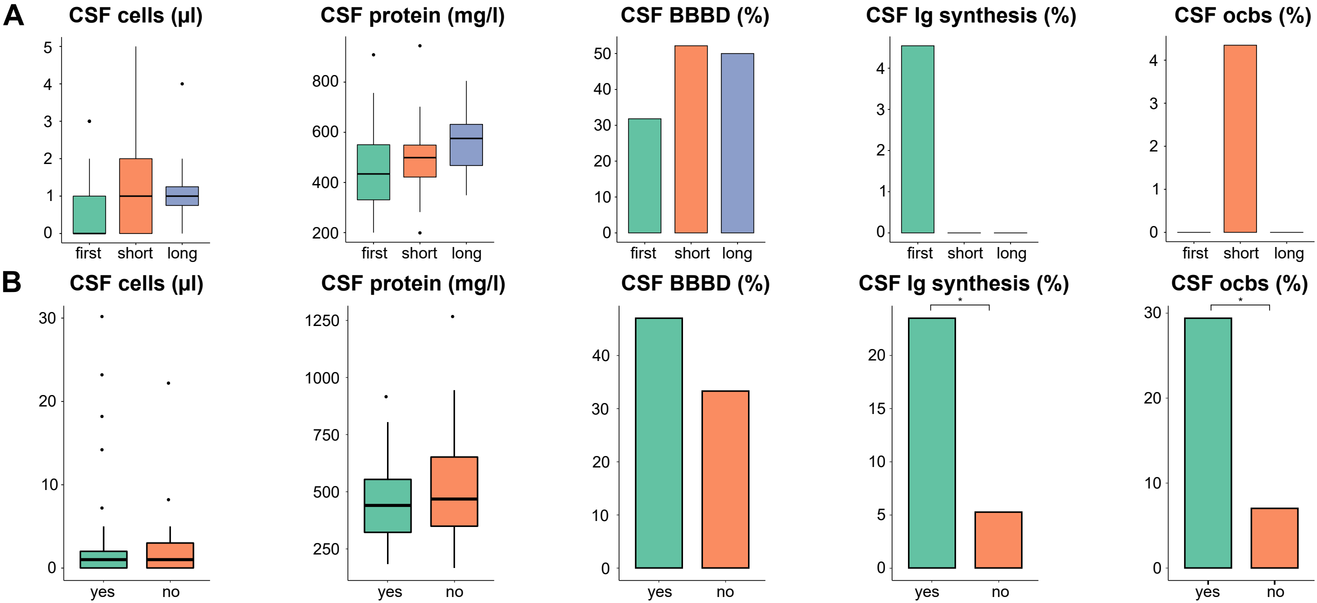

Supplementary figure 1: Comparison of basic CSF parameters between F2x patients with first diagnosis, with short disease course and with longstanding disease manifestation as well as between F2x patients with and without positive psychotic symptoms at time of sample taking

Basic CSF characteristics: cells were counted manually in a Fuchs-Rosenthal chamber; protein was assessed by nephelometry; BBBD was evaluated based on the serum/CSF albumin ratio; Ig synthesis was assessed by nephelometry; oligoclonal bands were detected by isoelectric focusing and silver nitrate staining. If the variables were continuous, significance was calculated using the two-sided Mann-Whitney U test for 2 groups and the Kruskal Wallis test with post hoc two-sided Dunn test. If the variables were continuous, we used the two-sided Fisher test to determine the significance. P values were adjusted for multiple hypothesis testing with Benjamini-Hochberg's procedure.

A Comparison of basic CSF parameters between F2x patients with first diagnosis, with short disease course and with longstanding disease manifestation;

B Comparison of basic CSF parameters between F2x patients with and without positive psychotic symptoms at time of sample taking

(BBBD: blood-brain-barrier dysfunction; CSF: cerebrospinal; first: first diagnosis; fluid; F2x: patients with psychotic disorder, Ig: immunoglobulin; IIH: intracranial hypertension; long: long disease course; NMDARE: anti-NMDA-receptor encephalitis; ocb: oligoclonal band; no: no positive psychotic symptoms at time of sample taking; RRMS: Relapsing-Remitting Multiple Sclerosis; yes: positive psychotic symptoms at time of sample taking)

Supplementary figure 2

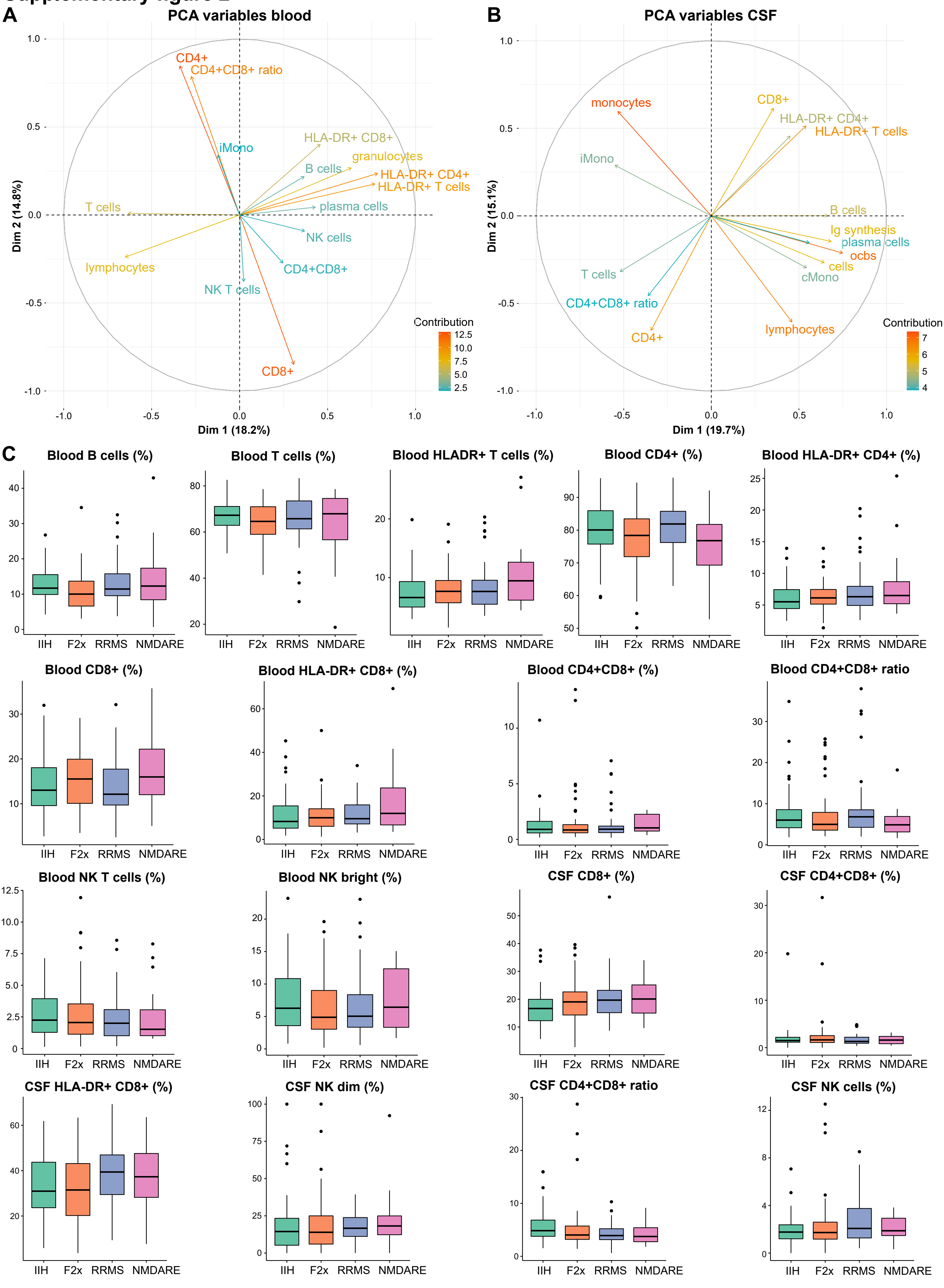

Supplementary figure 2: Variables contributing to PCA and additional boxplots of CSF and blood flow cytometry parameters

A Variables contributing to PCA of blood flow cytometry parameters. The 15 most important parameters are displayed. Coordinates of the variables are illustrated by circles. Contribution of variables to PC is presented by color. **B** Variables contributing to PCA of CSF basic and flow cytometry parameters. **C** Box plots of non-significant blood and CSF flow cytometry parameters: Lower quartile, median and upper quartile are shown by boxes. Whiskers depict 1.5 times the interquartile range of the box and outliers are illustrated by dots. The Kruskal Wallis tests with post hoc two-sided Dunn test and Benjamini-Hochberg's adjusted p values were used to calculate the significance.

(cMono: classical monocytes; Dim: dimension; F2x: patients with psychotic disorder; Ig: immunoglobulin; IIH: intracranial hypertension; iMono: intermediate monocytes; NK: natural killer cells; NKT: natural killer T cell; NMDARE: anti-NMDA-receptor encephalitis; ocb: oligoclonal band; PC: principal component; PCA: principal component analysis; RRMS: Relapsing-Remitting Multiple Sclerosis)

Supplementary figure 3

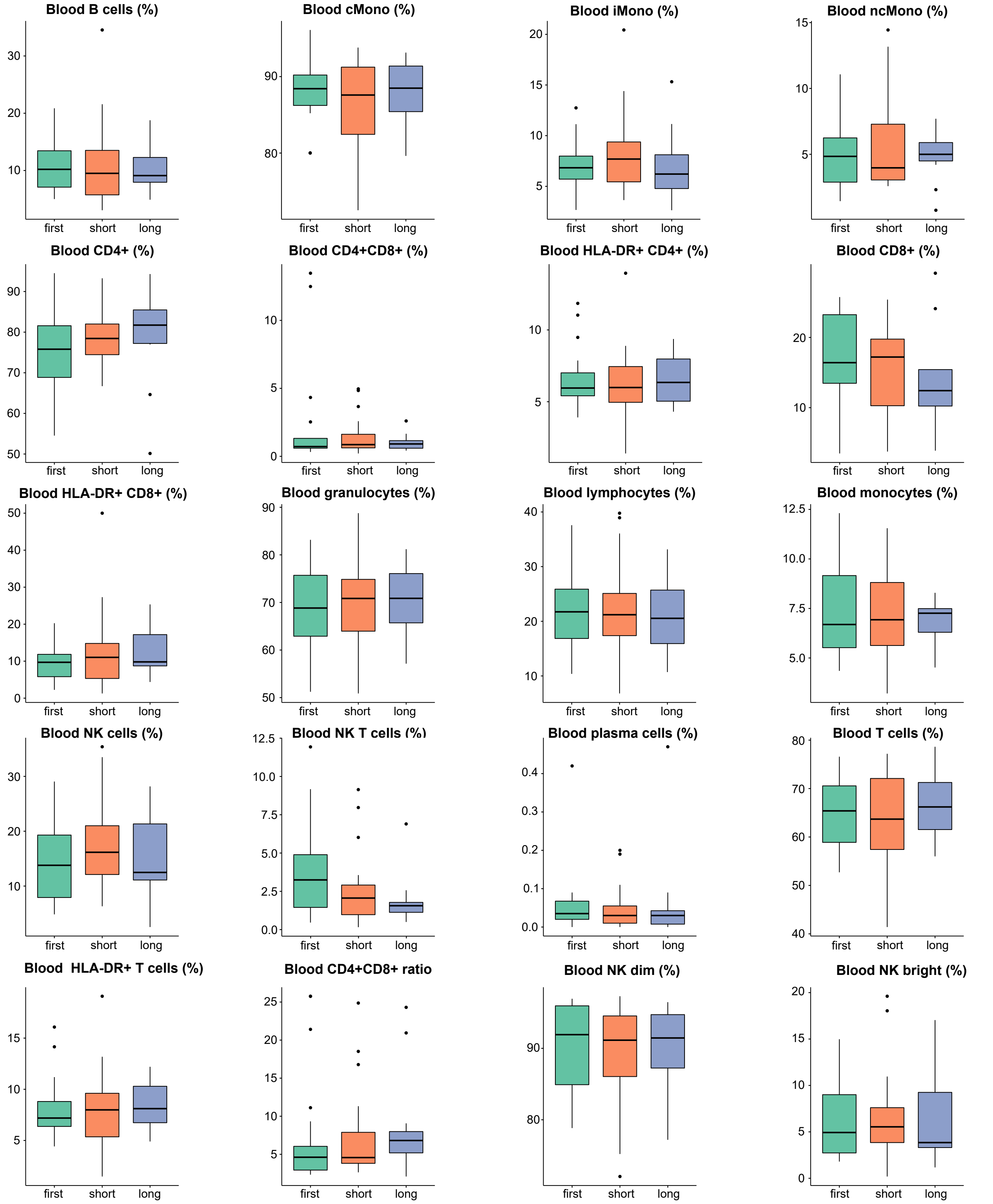

Supplementary figure 3: Comparison of blood flow cytometry parameters between F2x patients with first diagnosis, with short disease course and with longstanding disease manifestation

Box plots of selected individual blood flow cytometry parameters: Lower quartile, median and upper quartile are shown by boxes. Whiskers depict 1.5 times the interquartile range of the box and outliers are illustrated by dots. The Kruskal Wallis tests with post hoc two-sided Dunn test and Benjamini-Hochberg's adjusted p values were used to calculate the significance.

(cMono: classical monocytes; first: first diagnosis; F2x: patients with psychotic disorder; IIH: intracranial hypertension; iMono: intermediate monocytes; long: long disease course; ncMono: non-classical monocytes; NK: Natural Killer cells; ncMono: non-classical monocytes; NMDARE: anti-NMDA-receptor encephalitis; RRMS: Relapsing-Remitting Multiple Sclerosis; short: short disease course)

Supplementary figure 4

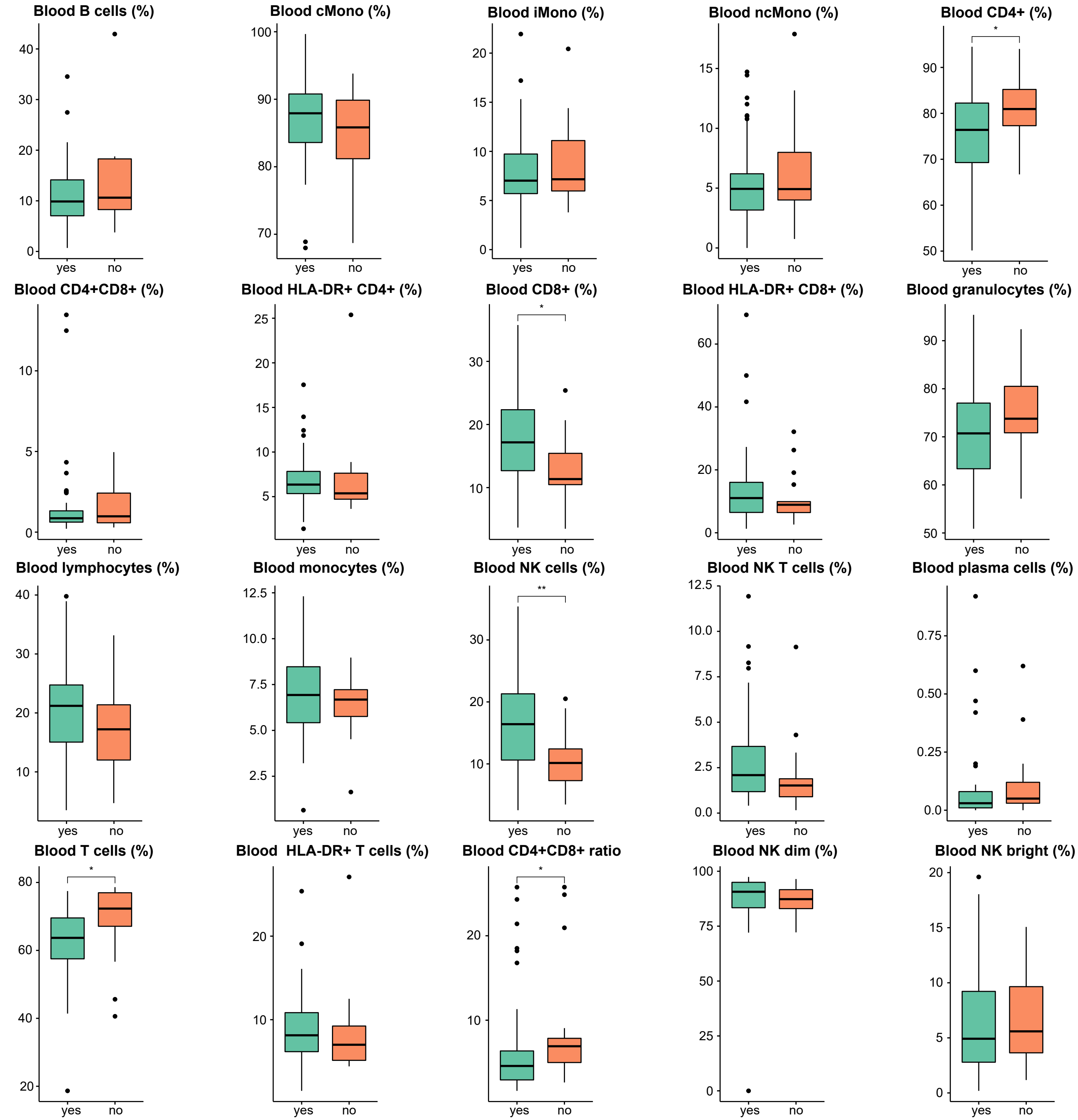

Supplementary figure 4: Comparison of blood flow cytometry parameters between F2x patients with and without positive psychotic symptoms at time of sample taking

Box plots of selected individual blood flow cytometry parameters: Lower quartile, median and upper quartile are shown by boxes. Whiskers depict 1.5 times the interquartile range of the box and outliers are illustrated by dots.The two-sided Mann-Whitney U test was used to calculate the significance.

(cMono: classical monocytes; F2x: patients with psychotic disorder; IIH: intracranial hypertension; iMono: intermediate monocytes; ncMono: non-classical monocytes; NK: natural killer cells; NMDARE: anti-NMDA-receptor encephalitis; no: no positive psychotic symptoms at time of sample taking; RRMS: Relapsing-Remitting Multiple Sclerosis; yes: positive psychotic symptoms at time of sample taking)

Supplementary figure 5

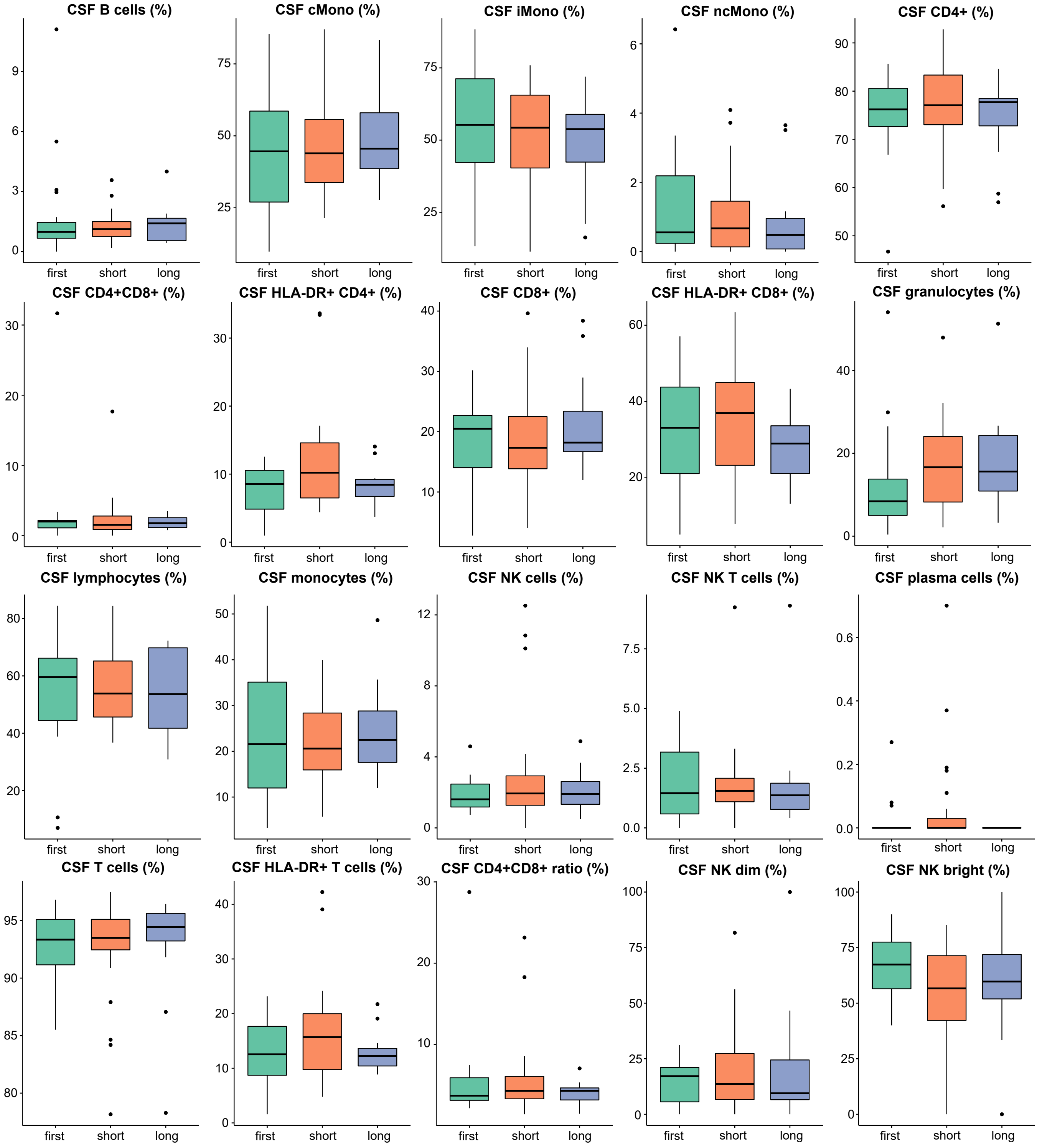

Supplementary figure 5: Comparison of CSF flow cytometry parameters between F2x patients with first diagnosis, with short disease course and with longstanding disease manifestation

Box plots of selected individual CSF flow cytometry parameters: Lower quartile, median and upper quartile are shown by boxes. Whiskers depict 1.5 times the interquartile range of the box and outliers are illustrated by dots. The Kruskal Wallis tests with post hoc two-sided Dunn test and Benjamini-Hochberg's adjusted p values were used to calculate the significance.

(cMono: classical monocytes; first: first diagnosis; Corr: correlation; F2x: patients with psychotic disorder; IIH: intracranial hypertension; iMono: intermediate monocytes; long: long disease course; ncMono: non-classical monocytes; NK: Natural Killer cells; NMDARE: anti-NMDA-receptor encephalitis; RRMS: Relapsing-Remitting Multiple Sclerosis; short: short disease course)

Supplementary figure 6

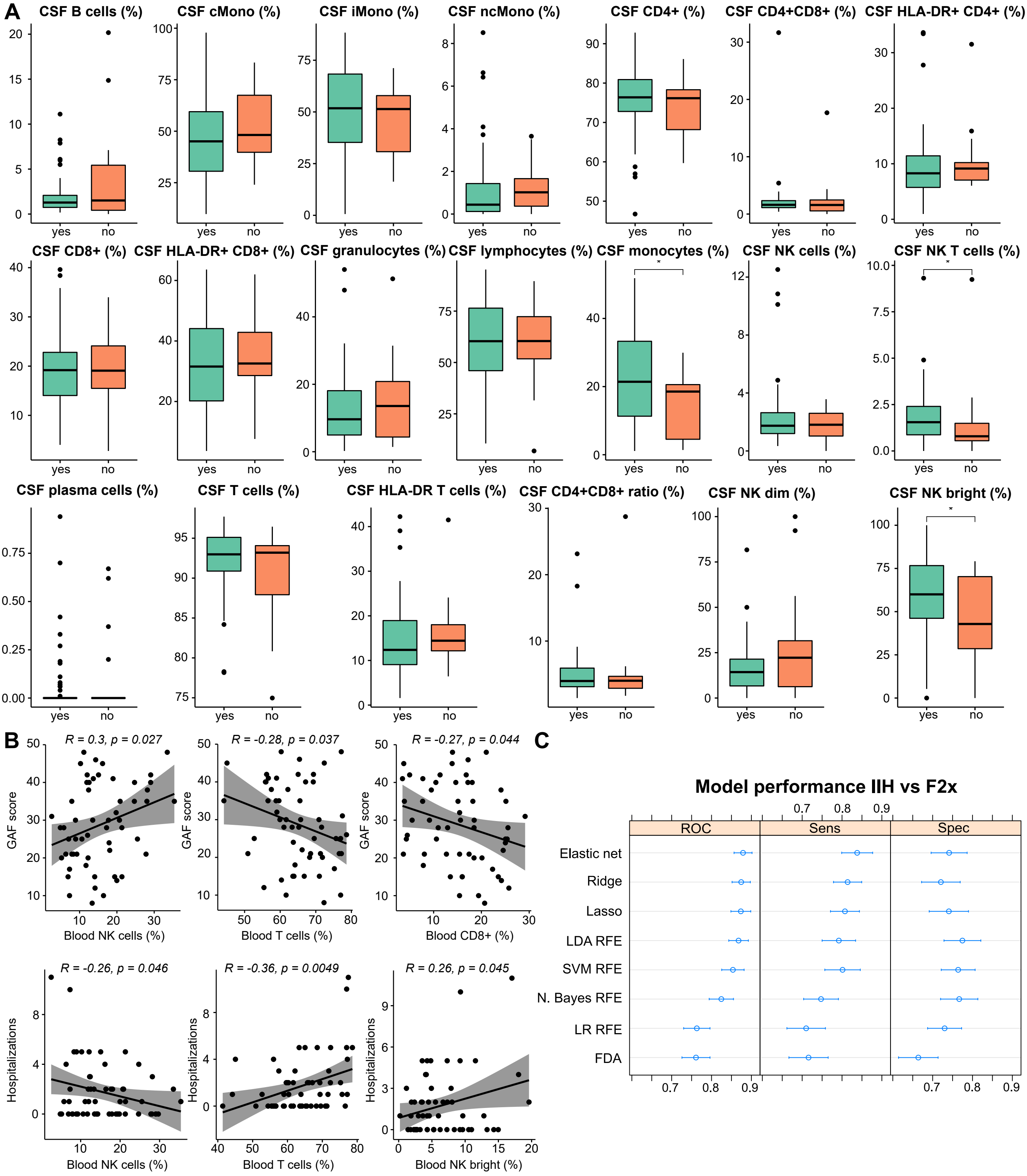

Supplementary figure 6: Comparison of CSF flow cytometry parameters between F2x patients with and without positive psychotic symptoms at time of sample taking and best performing models to differentiate F2x and controls

A Box plots of selected individual CSF flow cytometry parameters: Lower quartile, median and upper quartile are shown by boxes. Whiskers depict 1.5 times the interquartile range of the box and outliers are illustrated by dots. The two-sided Mann-Whitney U test was used to calculate the significance. **B** Correlation between individual blood/CSF flow cytometry parameters and GAF scores as well as hospitalizations due to psychotic symptoms. We performed linear regression analysis with the Pearson correlation coefficient (R). The grey areas show the confidence interval. **C** Model performance to distinguish F2x from IIH when including basic CSF and flow cytometry parameters. F2x was defined as positive and IIH as negative.

(cMono: classical monocytes; Corr: correlation; CSF: cerebrospinal fluid; FDA: flexible discriminant analysis; F2x: patients with psychotic disorder; GAF: Global Assessment of Functioning; Ig: immunoglobulin; IIH: intracranial hypertension; iMono: intermediate monocytes; Lasso: least absolute shrinkage and selection operator, LDA RFE: linear discriminant analysis with recursive feature elimination; LR RFE: logistic regression with recursive feature elimination; N. Bayes: naive bayes; ncMono: non-classical monocytes; NK: Natural killer cells; NMDARE: anti-NMDA-receptor encephalitis; no: no positive psychotic symptoms at time of sample taking; RFE: recursive feature elimination; ROC: receiver operating characteristic; RRMS: Relapsing-Remitting Multiple Sclerosis; yes: positive psychotic symptoms at time of sample taking; Sens: sensitivity; Spec: specificity; SVM: Support vector machines)
