# Supplementary material for: Cerebrospinal fluid flow cytometry distinguishes psychosis spectrum disorders from differential diagnoses
Source: Mol Psychiatry. 2021 Aug 6;26(12):7661–70. doi: 10.1038/s41380-021-01244-5 (PMC8873003; doi:10.1038/s41380-021-01244-5)
Supplement: Supplementary file 2 — Supplementary tables [file 41380_2021_1244_MOESM2_ESM.pdf]

Supplementary table 1

Group	Subgroup	Pseudonym	Sex	Age	Clinical signs (at sample taking)	Pos. psychotic sympt. (a.s.t.)	Disease duration (years)	Prev. hosp.	GAF	APDs	Comorb. (a.s.t.)	NMDAR ab	ICU (a.s.t.)
F2x	F20.0	261630	m	35	hallucinations, delusions	yes	0	0	32	1	arterial hypertension	no	no
F2x	F20.0	329163	m	23	paranoid delusions, hallucinations	yes	0	1	28	1	conjunctivitis	no	no
F2x	F20.0	337421	m	22	visual hallucinations, paranoid delusions, social withdrawal	yes	4	4	45	1	none	no	no
F2x	F20.0	493172	m	45	chronic acoustic hallucinations, poor attention	yes	8	4	40	1	hyperthyroidism	no	no
F2x	F20.0	481349	m	31	persistent acoustic hallucinations, delusions, sleep disturbance	yes	5	2	30	1	oesophagitis	no	no
F2x	F20.0	229838	f	27	paranoid delusions, delusions of grandeur	yes	4	2	10	1	conjunctivitis	no	no
F2x	F20.0	560933	f	28	paranoid delusions, cognitive deficits	yes	0	0	30	1	alcohol abuse, cannabis abuse, amphetamine abuse	no	no
F2x	F20.0	134672	m	24	desorganized behavior, delusions, obsessive-compulsive behaviour	yes	4	4	21	1	arthritis	no	no
F2x	F20.0	657447	m	23	chronic acoustic hallucinations, mood disturbances, impulse control deficits	yes	2	0	21	1	none	no	no
F2x	F20.0	257239	f	20	acute psychotic exacerbation with paranoid delusions, anxiety, aggressive behavior	yes	0	0	15	1	none	no	no
F2x	F20.0	628721	m	31	paranoid delusions, anxiety, disorganized behavior	yes	0	0	35	1	none	no	no
F2x	F20.0	162186	f	55	paranoid delusions, social withdrawal, blunted affect	yes	3	0	24	1	arterial hypertension	no	no
F2x	F20.0	394476	m	26	acoustic hallucinations, delusions, mood changes, anxiety	yes	3	2	40	1	none	no	no
F2x	F20.0	645563	m	22	delusions, depressive symptoms	yes	0	1	25	1	none	no	no
F2x	F20.0	727870	m	24	conversion disorder with motor symptoms	no	3	5	45	1	conversion disorder with motor symptom	no	no
F2x	F20.0	824899	m	28	paranoid delusions, social withdrawal, blunted affect	yes	0	0	21	1	cannabis abuse, Crohn's Disease	no	no
F2x	F20.0	831603	m	49	suicidal ideation, chronic acoustic hallucinations	yes	30	>10	31	1	none	no	no
F2x	F20.0	970549	f	22	depersonalization, derealization, attention deficit	no	1	1	25	0	cannabis abuse, borderline personality disorder	no	no
F2x	F20.0	567584	m	25	delusions, formal thought disorder, attention deficit, obsessions	yes	7	3	38	1	OCD, arterial hypertension	no	no
F2x	F20.0	320776	f	46	altered perceptions	no	8	1	42	1	conversion disorder, migraine	no	no
F2x	F20.0	224222	m	32	suicidal ideation, acoustic and visual hallucinations, delusions	yes	na	0	18	1	cannabis abuse	no	no
F2x	F20.0	349460	m	36	paranoid delusions, acoustic hallucinations	yes	11	5	28	1	none	no	no
F2x	F20.0	914975	m	18	paranoid delusions, illusions	yes	0	0	20	0	none	no	no
F2x	F20.0	255817	m	26	depressive symptoms and chronic acoustic hallucinations	yes	1	1	41	0	cannabis abuse	no	no
F2x	F20.1	998918	m	22	desorganized behavior, formal thought disorder	no	3	5	25	1	none	no	no
F2x	F20.1	631319	m	19	blunted affect, formal thought disorder, attention deficit	no	10	5	21	1	none	no	no
F2x	F20.2	564805	f	62	pernicious catatonia, ECT treatment, fluctuating consciousness	no	4	2	8	1	psoriasis	no	yes
F2x	F20.3	785071	m	45	paranoid delusions, desorganized behavior, tension	yes	4	3	20	1	alcohol abuse, arterial hypertension, obesity	no	no
F2x	F20.4	259990	f	54	depressive symptoms, social withdrawal	no	0	0	41	1	none	no	no
F2x	F22.0	417762	f	47	delusion, tactile hallucinations	yes	2	2	48	1	nodular goiter	no	no
F2x	F22.0	581234	m	37	delusions, anxiety, depression	yes	0	0	35	1	psoriasis	no	no
F2x	F22.0	570989	m	62	paranoid delusions, delusions of reference	yes	1	1	48	1	none	no	no
F2x	F22.0	423680	f	58	delusions, delusions of control	yes	4	0	na	1	migraine	no	no
F2x	F22.0	283220	m	60	paranoid delusions, delusions of control, acoustic hallucinations	yes	1	0	46	1	none	no	no
F2x	F22.0	170216	m	47	paranoid delusions	yes	1	0	40	1	none	no	no
F2x	F23.0	570688	m	58	paranoid delusions, agitation, anxiety	yes	0	0	28	1	stroke, myocardial infarction	no	no
F2x	F23.0	567263	m	25	paranoid delusions, formal thought disorder, suspiciousness	yes	0	0	28	1	none	no	no
F2x	F23.0	605522	f	31	delusions of control, formal thought disorder, agitation, anxiety	yes	0	0	30	0	none	no	no
F2x	F23.1	478219	f	47	paranoid delusions, derealization	yes	0	0	21	1	none	no	no
F2x	F23.1	789818	f	27	anxiety, paranoid delusions	yes	0	0	15	1	none	no	no
F2x	F23.1	446456	f	24	paranoid delusions, coenesthetic hallucinations	yes	0	0	35	1	none	no	no
F2x	F25.0	878131	m	20	manic-psychotic presentation with paranoid delusions and delusions of grandeur, agitation and impulsivity	yes	2	1	14	1	none	no	no
F2x	F25.1	705596	f	53	suicidality, severe depression	no	30	10	10	1	seizure	no	no
F2x	F25.1	479845	f	61	depressed mood, reduced energy	no	14	3	38	1	arterial hypertension	no	no
F2x	F25.1	236253	f	62	dysphoric mood, agitation, desorientation	no	12	5	26	1	type 2 diabetes, primary biliary cirrhosis	no	no
F2x	F25.1	460296	f	64	desorganized behavior, delusions, depressed mood	yes	1	2	30	1	cerebral microangiopathy	no	no
F2x	F25.1	144757	m	24	delusions of control, paranoid delusions, suicidal ideation	yes	2	1	35	1	arterial hypertension	no	no
F2x	F25.1	614608	f	44	somatic delusions, blunted affect, anhedonia	yes	5	5	42	1	seizures	no	no
F2x	F25.1	264491	m	45	delusions, high levels of anxiety, suicide attempt	yes	7	1	15	1	none	no	no
F2x	F25.1	544317	f	22	depressed mood, reduced energy, rumination, anxiety	yes	1	0	na	1	PCO, neurodermatitis	no	no
F2x	F25.2	512581	m	66	depressed mood, paranoid delusions, formal thought disorder	yes	55	2	35	1	atrial fibrillation	no	no
F2x	F23.2	122988	f	31	paranoid delusions, acoustic hallucinations	yes	0	0	25	1	none	no	no
F2x	F25.2	611704	m	23	delusions of control, somatic delusions	yes	0	0	42	1	hypthyroidism	no	no
F2x	F20.0	660254	m	25	suicidality, thought insertion, reduced energy	yes	0.5	2	22	1	none	no	no
F2x	F20.0	763828	m	22	concentration deficits, thought insertion	yes	0.5	1	45	1	none	no	no
F2x	F20.0	655091	m	29	delusions of reference, paranoid delusions, concentration deficits	yes	0.5	2	17	1	none	no	no
F2x	F20.0	953835	m	40	depressed mood, reduced energy, concentration deficits	no	17	2	40	1	amphetamine abuse, ADHD	no	no
F2x	F23.1	377589	m	22	delusions of grandeur, thought withdrawal, formal thought disorder	yes	0	0	12	1	none	no	no
F2x	F20.0	646918	f	34	paranoid delusions, visual hallucinations, delusions of control	yes	0	0	15	1	none	no	no
NMDARE	-	975938	f	37	acute psychosis	yes	-	-	-	-	teratoma	yes	no
NMDARE	-	679603	f	22	seizures	no	-	-	-	-	none	yes	no
NMDARE	-	571459	f	18	cognitive impairment, personality changes, hallucinations, avolition	yes	-	-	-	-	none	yes	no
NMDARE	-	969801	m	78	seizures, altered mental status, dysphagia	no	-	-	-	-	s/p EBV encephalitis, OSA, diabetes, arterial hypertension	yes	no
NMDARE	-	787718	f	22	cognitive impairment, speech disturbance, anxiety, hallucinations	yes	-	-	-	-	neurodermatitis, migraine, tension headache	yes	no
NMDARE	-	660736	m	42	psychomotor redardation, personality changes, speech disturbance, fever, myoclonia, altered mental status, dysphagia, seizures	no	-	-	-	-	none	yes	no
NMDARE	-	208806	f	51	disorientation, agitation, psychomotor retardation	no	-	-	-	-	atrial fibrillation, SCC, diabetes	yes	no
NMDARE	-	340667	m	25	personality changes, acute psychosis, aggressive behaviour, mutism, stupor, seizures	yes	-	-	-	-	none	yes	no
NMDARE	-	435039	f	68	hallucinations	yes	-	-	-	-	arterial hypertension, hypothyroidism	yes	no
NMDARE	-	417259	f	28	acute psychosis, seizures	yes	-	-	-	-	none	yes	no
NMDARE	-	477294	f	21	personality changes, speech disturbance, disorientation, psychomotor retardation, emotional instability, cognitive impairment, avolition	yes	-	-	-	-	hypothyroidism, migraine, tension headache	yes	no
NMDARE	-	992432	f	18	insomnia, acute psychosis, seizures, mutism, hypersexuality	yes	-	-	-	-	teratoma	yes	na
NMDARE	-	391845	f	26	fever, seizures	no	-	-	-	-	none	yes	no
NMDARE	-	411123	f	23	insomnia, cognitive impairment, psychomotor retardation	no	-	-	-	-	none	yes	no
NMDARE	-	518953	f	47	disorientation, altered mental status, cognitive impairment, avolition, fever, hallucinations	yes	-	-	-	-	s/p HSV encephalitis	yes	no
NMDARE	-	219839	m	66	acute psychosis	yes	-	-	-	-	none	yes	no

Supplementary table 1: Additional clinical characteristics of F2x and NMDARE patients

Ab: antibody; ADHD: attention deficit hyperactivity disorder; APDs: antipsychotic drugs; A.s.t.: at sample taking; Comorb: comorbidities; CSF - cerebrospinal fluid; ICU - Intensive Care Unit; f: female; DD: differential diagnosis; Dx: diagnosis; EBV: Epstein–Barr virus; ECT - electroconvulsive therapy; F2x: patients with psychotic disorder; GAF: Global Assessment of Functioning; Hosp: hospitalizations; HSV: Herpes simplex virus; NMDARE: anti-NMDA-receptor encephalitis; m: male; OCD - obsessive-compulsive disorder; OSA - Obstructive sleep apnea; PCO - polycystic ovary syndrome; Pos: positive; Prev: previous; Pseud: pseudonym; SCC - squamous cell carcinoma; s/p: status post; sympt: symptoms

Supplementary table 2

	Outcome	Term	Estimate	Std.error	Statistic	P-value
**F2x vs IIH**	Blood cMono	dxF2x	2.5359	1.2786	1.9834	0.0489
	Blood iMono	dxF2x	-4.0791	0.9967	-4.0926	0.0001
	Blood monocytes	dxF2x	0.5532	0.4895	1.1301	0.2601
	Blood NK cells	dxF2x	4.0075	1.4729	2.7209	0.0072
	CSF protein	dxF2x	65.9218	29.6529	2.2231	0.0275
	CSF ncMono	dxF2x	0.7723	0.3674	2.1019	0.0370
	CSF CD4+	dxF2x	-3.4326	1.7549	-1.9560	0.0521
	CSF lymphocytes	dxF2x	-8.8637	3.3703	-2.6300	0.0093
	CSF monocytes	dxF2x	4.2888	2.0287	2.1140	0.0360
	CSF BBBD	dxF2x	0.2181	0.0785	2.7779	0.0061
**F2x vs RRMS**	Blood plasma cells	dxF2x	-0.0222	0.0222	-1.0016	0.3180
	CSF cells	dxF2x	-6.9370	1.2825	-5.4091	0.0000
	CSF BBBD	dxF2x	0.1889	0.0768	2.4611	0.0149
	CSF Ig synthesis	dxF2x	-0.5720	0.0617	-9.2698	0.0000
	CSF ocbs	dxF2x	-0.8528	0.0493	-17.2878	0.0000
	CSF B cells	dxF2x	-2.9297	0.5290	-5.5384	0.0000
	CSF cMono	dxF2x	-9.8327	3.7565	-2.6175	0.0097
	CSF iMono	dxF2x	9.6811	3.7150	2.6060	0.0100
	CSF HLA-DR+CD4+	dxF2x	-2.7357	1.1469	-2.3853	0.0182
	CSF granulocytes	dxF2x	4.3651	2.1996	1.9845	0.0488
	CSF lymphocytes	dxF2x	-20.5043	3.2945	-6.2237	0.0000
	CSF monocytes	dxF2x	13.8677	1.9831	6.9928	0.0000
	CSF NK cells	dxF2x	0.8192	0.3009	2.7221	0.0072
	CSF plasma cells	dxF2x	-0.6575	0.1128	-5.8275	0.0000
	CSF T cells	dxF2x	2.4631	0.9007	2.7346	0.0069
	CSF HLA-DR+T cells	dxF2x	-3.8443	1.4685	-2.6179	0.0097
**F2x vs NMDARE**	Blood cMono	dxF2x	4.4450	1.8010	2.4680	0.0146
	Blood iMono	dxF2x	-3.0283	1.4040	-2.1570	0.0324
	Blood granulocytes	dxF2x	-7.1205	2.4714	-2.8811	0.0045
	Blood lymphocytes	dxF2x	6.5321	2.1188	3.0829	0.0024
	Blood plasma cells	dxF2x	-0.1633	0.0320	-5.1086	0.0000
	Blood NK dim	dxF2x	10.3580	2.7551	3.7595	0.0002
	CSF cells	dxF2x	-7.9329	1.8481	-4.2926	0.0000
	CSF protein	dxF2x	37.4051	41.7697	0.8955	0.3718
	CSF BBBD	dxF2x	0.2170	0.1106	1.9618	0.0514
	CSF Ig synthesis	dxF2x	-0.3368	0.0889	-3.7875	0.0002
	CSF ocbs	dxF2x	-0.4756	0.0711	-6.6906	0.0000
	CSF B cells	dxF2x	-4.8341	0.7623	-6.3418	0.0000
	CSF cMono	dxF2x	-16.0706	5.4131	-2.9688	0.0034
	CSF iMono	dxF2x	17.7624	5.3533	3.3180	0.0011
	CSF granulocytes	dxF2x	9.0018	3.1697	2.8400	0.0051
	CSF lymphocytes	dxF2x	-23.1083	4.7474	-4.8675	0.0000
	CSF monocytes	dxF2x	12.6982	2.8577	4.4435	0.0000
	CSF plasma cells	dxF2x	-0.1463	0.1626	-0.8999	0.3694
	CSF T cells	dxF2x	3.3017	1.2980	2.5438	0.0119
	CSF NK bright	dxF2x	17.6142	5.7973	3.0384	0.0028
**F2x corr. GAF**	CSF ncMono	GAF	-0.0451	0.0201	-2.2456	0.0290
	Blood CD 8+	GAF	-0.1793	0.0844	-2.1227	0.0386
	Blood NK cells	GAF	0.2052	0.0990	2.0725	0.0432
	Blood T cells	GAF	-0.2249	0.1018	-2.2091	0.0316
**F2x corr. hospitalizations**	CSF protein	prev. hosp.	13.1021	8.5662	1.5295	0.1320
	CSF NK T cells	prev. hosp.	0.1958	0.0996	1.9667	0.0544
	Blood NK cells	prev. hosp.	-0.9537	0.4441	-2.1473	0.0363
	Blood T cells	prev. hosp.	1.3856	0.4705	2.9453	0.0048
	Blood NK bright	prev. hosp.	0.5113	0.2518	2.0303	0.0473
**F2x corr. duration**	CSF protein	disease duration	3.8572	2.3948	1.6107	0.1132
	Blood iMono	disease duration	0.1035	0.0525	1.9703	0.0540

Supplementary table 2: Multiple regression analysis adjusting for age and sex

BBBD: blood-brain-barrier dysfunction; cMono: classical monocytes; corr: correlation; CSF: cerebrospinal fluid; dx: diagnosis; F2x: patients with psychotic disorder; hosp: hospitalizations; Ig: immunoglobulin; IIH: intracranial hypertension; iMono: intermediate monocytes; ncMono: non-classical monocytes; NK: natural killer cells; NMDARE: anti-NMDA-receptor encephalitis; ocb: oligoclonal band; p: previous; RRMS: Relapsing-Remitting Multiple Sclerosis; Std: standard; vs: versus

Supplementary table 3

F2x vs IIH (blood)

Parameter	AUC
iMono	0.7807
NK cells	0.6741
Monocytes	0.6636
cMono	0.6620
B cells	0.6456
ncMono	0.6154
T cells	0.5957
NK dim	0.5792
HLA-DR+CD4+	0.5764
Granulocytes	0.5736
CD4+	0.5713
NK bright	0.5660
CD4+CD8+ ratio	0.5656
HLA-DR+ T cells	0.5642
CD 8+	0.5597
NK T cells	0.5371
Lymphocytes	0.5252
HLA-DR+CD8+	0.5184
CD4+CD8+	0.5078
Plasma cells	0.4654

F2x vs IIH (CSF)

Parameter	AUC
Protein	0.7362
ncMono	0.7172
Lymphocytes	0.6752
Monocytes	0.6403
CD4+	0.6346
CD4+CD8+ ratio	0.6170
CD8+	0.6139
B cells	0.5860
T cells	0.5597
Nk T cells	0.5549
Granulocytes	0.5499
iMono	0.5441
CD4+CD8+	0.5345
NK bright	0.5335
cMono	0.5308
HLA-DR+ T cells	0.5167
HLA-DR+CD4+	0.5096
cells	0.5010
NK dim	0.4995
HLA-DR+CD8+	0.4949
Ig synthesis	0.4914
Ocbs	0.4914
Plasma cells	0.4819
NK cells	0.4753
BBBD	0.3326

F2x vs RRMS (blood)

Parameter	AUC
B cells	0.6195
Monocytes	0.6191
Plasma cells	0.6191
NK cells	0.6087
Granulocytes	0.6034
iMono	0.6017
Lymphocytes	0.5911
CD4 +	0.5822
CD4+CD8+ ratio	0.5767
cMono	0.5695
CD8+	0.5672
T cells	0.5626
NK T cells	0.5447
HLA-DR+CD4+	0.5232
HLA-DR+ T cells	0.5048
ncMono	0.5023
CD4 + CD8 +	0.5023
NK dim	0.4937
NK bright	0.4898
HLA-DR+CD8+	0.4761

F2x vs RRMS (CSF)

Parameter	AUC
Ocbs	0.9302
Plasma cells	0.8967
Monocytes	0.8804
Lymphocytes	0.8293
Cells	0.8024
Ig synthesis	0.7975
B cells	0.7964
NK T cells	0.7634
iMono	0.6828
cMono	0.6793
Granulocytes	0.6740
HLA-DR+ T cells	0.6564
HLA-DR+CD4+	0.6473
T cells	0.6429
HLA-DR+CD8+	0.6047
Protein	0.5901
CD4+CD8+	0.5827
NK cells	0.5760
NK dim	0.5574
ncMono	0.5533
NK bright	0.5470
CD4+	0.5271
CD8+	0.5264
CD4+CD8+ ratio	0.5260
BBBD	0.3661

F2x vs NMDARE (blood)

Parameter	AUC
Plasma cells	0.7834
Lymphocytes	0.7505
NK dim	0.7462
iMono	0.7414
Granulocytes	0.7360
cMono	0.7107
NK cells	0.6347
HLA-DR+ T cells	0.6078
B cells	0.5981
NK bright	0.5927
HLA-DR+CD8+	0.5916
Monocytes	0.5787
CD4+CD8+ ratio	0.5787
CD8+	0.5776
ncMono	0.5749
CD4+	0.5636
CD4+CD8+	0.5593
HLA-DR+CD4+	0.5593
T cells	0.5506
NK T cells	0.5242

F2x vs NMDARE (CSF)

Parameter	AUC
Lymphocytes	0.8707
Monocytes	0.8438
B Cells	0.8206
Cells	0.8023
Granulocytes	0.8006
iMono	0.7656
cMono	0.7441
Ocbs	0.7414
Plasma cells	0.7101
Protein	0.7047
NK bright	0.7037
T cells	0.6832
Ig synthesis	0.6789
NK T cells	0.6730
HLA-DR+CD8+	0.5894
ncMono	0.5695
CD4+CD8+	0.5668
CD4+	0.5587
CD8+	0.5560
HLA-DR+ T cells	0.5560
NK dim	0.5550
CD4+CD8+ ratio	0.5528
NK cells	0.5436
HLA-DR+CD4+	0.5323
BBBD	0.3470

Supplementary table 3: ROC AUC values of F2x vs IIH, RRMS, and NMDARE patients

AUC: area under the curve; BBBD: blood-brain-barrier dysfunction; cMono: classical monocytes; CSF: cerebrospinal fluid; F2x: patients with psychotic disorder; Ig: immunoglobulin; IIH: intracranial hypertension; iMono: intermediate monocytes; ncMono: non-classical monocytes; NK: natural killer cells; NMDARE: anti-NMDA-receptor encephalitis; ocb: oligoclonal band; RRMS: Relapsing-Remitting Multiple Sclerosis.

Supplementary table 4

F2x vs IIH (combined)	Model	ROC	ROC SD	Sens	Sens SD	Spec	Spec SD
	**Elastic net**	0.87902	0.07939	0.83800	0.13653	0.74133	0.15887
	**FDA**	0.76098	0.12374	0.71600	0.17317	0.66400	0.17455
	**Lasso**	0.87351	0.08755	0.80733	0.12765	0.74067	0.17331
	**LDA RFE**	0.86782	0.08644	0.79200	0.14778	0.77467	0.16254
	**LR RFE**	0.76309	0.11626	0.71000	0.17000	0.73000	0.15002
	**N. Bayes RFE**	0.82456	0.10707	0.74733	0.15343	0.76667	0.16454
	**Ridge**	0.87442	0.08055	0.81400	0.12437	0.72067	0.17035
	**SVM RFE**	0.85362	0.09873	0.80133	0.15649	0.76400	0.14950

Supplementary table 4: Performance of multiparametric models to distinguish F2x from control patients

FDA: flexible discriminant analysis; F2x: patients with psychotic disorder; IIH: intracranial hypertension; Lasso: least absolute shrinkage and selection operator, LDA RFE: linear discriminant analysis with recursive feature elimination; LR RFE: logistic regression with recursive feature elimination; N. Bayes: naive bayes; RFE: recursive feature elimination; ROC: receiver operating characteristic; SD: standard deviation; Sens: sensitivity; Spec: specificity; SVM: support vector machines
